# Gene Expansion and Positive Selection as Bacterial Adaptations to Oligotrophic Conditions

**DOI:** 10.1128/mSphereDirect.00011-19

**Published:** 2019-02-06

**Authors:** Ruben Props, Pieter Monsieurs, Peter Vandamme, Natalie Leys, Vincent J. Denef, Nico Boon

**Affiliations:** a Center for Microbial Ecology and Technology (CMET), Ghent University, Ghent, Belgium; b Belgian Nuclear Research Centre (SCK•CEN), Mol, Belgium; c Department of Ecology and Evolutionary Biology, University of Michigan, Ann Arbor, Michigan, USA; d Laboratory of Microbiology, Faculty of Sciences, Ghent University, Ghent, Belgium; e Unit Health, Flemish Institute for Technological Research (VITO), Mol, Belgium; Department of Energy Joint Genome Institute; University of Zurich; Uppsala University

**Keywords:** genomic adaptations, microbial ecology, oligotrophy, selection, streamlining

## Abstract

We examined the genomic adaptations of prevalent bacterial taxa in a highly nutrient- and ion-depleted freshwater environment located in the secondary cooling water system of a nuclear research reactor. Using genome-centric metagenomics, we found that none of the prevalent bacterial taxa were related to typical freshwater bacterial lineages. We also did not identify strong signatures of genome streamlining, which has been shown to be one of the ecoevolutionary forces shaping the genome characteristics of bacterial taxa in nutrient-depleted environments. Instead, focusing on the dominant taxon, a novel *Ramlibacter* sp. which we propose to name Ramlibacter aquaticus, we detected extensive positive selection on genes involved in phosphorus and carbon scavenging pathways. These genes were involved in the high-affinity phosphate uptake and storage into polyphosphate granules, metabolism of nitrogen-rich organic matter, and carbon/energy storage into polyhydroxyalkanoate. In parallel, comparative genomics revealed a high number of paralogs and an accessory genome significantly enriched in environmental sensing pathways (i.e., chemotaxis and motility), suggesting extensive gene expansions in R. aquaticus. The type strain of *R. aquaticus* (LMG 30558^T^) displayed optimal growth kinetics and productivity at low nutrient concentrations, as well as substantial cell size plasticity. Our findings with *R. aquaticus* LMG 30558^T^ demonstrate that positive selection and gene expansions may represent successful adaptive strategies to oligotrophic environments that preserve high growth rates and cellular productivity.

**IMPORTANCE** By combining a genome-centric metagenomic approach with a culture-based approach, we investigated the genomic adaptations of prevalent populations in an engineered oligotrophic freshwater system. We found evidence for widespread positive selection on genes involved in phosphorus and carbon scavenging pathways and for gene expansions in motility and environmental sensing to be important genomic adaptations of the abundant taxon in this system. In addition, microscopic and flow cytometric analysis of the first freshwater representative of this population (*Ramlibacter aquaticus* LMG 30558^T^) demonstrated phenotypic plasticity, possibly due to the metabolic versatility granted by its larger genome, to be a strategy to cope with nutrient limitation. Our study clearly demonstrates the need for the use of a broad set of genomic tools combined with culture-based physiological characterization assays to investigate and validate genomic adaptations.

## Abstract (precis)

By combining a genome-centric metagenomic approach with a culture-based approach, we investigated the genomic adaptations of prevalent populations in an engineered oligotrophic freshwater system. We found evidence for widespread positive selection on genes involved in phosphorus and carbon scavenging pathways and for gene expansions in motility and environmental sensing to be important genomic adaptations of the abundant taxon in this system. In addition, microscopic and flow cytometric analysis of the first freshwater representative of this population (*Ramlibacter aquaticus* LMG 30558^T^) demonstrated phenotypic plasticity, possibly due to the metabolic versatility granted by its larger genome, to be a strategy to cope with nutrient limitation. Our study clearly demonstrates the need for the use of a broad set of genomic tools combined with culture-based physiological characterization assays to investigate and validate genomic adaptations.

## INTRODUCTION

Nutrient (co)limitation is one of the primary constraints of bacterial productivity in aquatic ecosystems (1–3) and has been shown to shape the bacterioplankton community composition (4, 5). As such, nutrient depletion and limitation play a crucial role in regulating biogeochemical processes (6). In recent years, (meta)genomic analyses have allowed *in situ* probing of relationships between environmental drivers and the genome characteristics of bacterial taxa (7–10). The genomic properties (e.g., %GC, genome size) of cooccurring taxa can vary considerably, which has been hypothesized to be the result of distinct ecoevolutionary mechanisms that act on each of the constituent taxa (11, 12). For example, nitrogen and carbon limitations have been correlated with genome size, GC content, and carbon/nitrogen protein content (13), thermal adaptation with genome size (8), and growth rate with GC content and codon usage bias (14, 15). These observations are strengthened by evidence for widespread selection on the GC content of bacteria (16), although the mechanism of GC-biased gene conversion that specifically acts on recombining genomic regions has also been postulated to play a significant role in shaping genomic GC content (17). Genes can, driven by natural selection, be adaptively changed by positive selection for advantageous mutations. Patterns of (genome-wide) positive selection have revealed lineage-specific adaptations, and together with codon usage bias, are thought to mediate fine-tuning of gene expression (18–20). Positive selection has been shown to be active in the adaptation of some bacterioplankton populations (*Polynucleobacter* sp.), although it was complemented with habitat-specific differences in functional gene content among the closely related populations (21).

Genome streamlining theory has successfully described the genomic adaptations of several abundant bacterioplankton groups that are characterized by small genome sizes and often proliferate in nutrient-limited environments (e.g., SAR11 lineage [22, 23], freshwater *Actinobacteria* and *Polynucleobacter* [24, 25], and subsurface *Archaea* [26]). These taxa gain a fitness advantage by possessing small cell (≤ ∼1 µm) and genome sizes (∼1 to 2 Mbp), high coding densities (> ∼90%), conserved core genes, and few pseudo- and paralog genes (10, 27). While carbon and nitrogen limitations have been strongly associated with genome streamlining (13), the effect of phosphorus limitation is uncertain (10, 28). Other genomic adaptations, such as those of bacterioplankton populations with relatively large genomes (e.g., > ∼3 to 4 Mbp) are less clear. They may have been the outcome of lineage-specific gene expansions, caused by gene duplication or lateral gene transfer events, which have been shown to account for up to 33% of available coding capacity in a diverse set of 21 isolate genomes (29). A meta-analysis of 115 large bacterial genomes demonstrated that the number of ABC transporter genes increased proportionally with genome size and that larger genomes are enriched in genes involved in regulation, secondary metabolism, and energy metabolism (11). It has also been postulated that taxa carrying such genomes are adapted to environments where competition for diverse resources is the primary selective pressure (11) and that despite their larger genome size, some of these genomes may still be subjected to streamlining (10).

Here, we examined whether these different genomic adaptations (i.e., genome streamlining, positive selection on core gene sequences, and/or gene expansion) have facilitated the success of prevalent bacterial taxa in an engineered oligotrophic freshwater environment. We studied a secondary cooling water system operating on a nuclear test reactor, for which its nutrient and carbon concentrations were tightly controlled by ion removal unit processes (conductivity of <7 µS cm^−1^). This system was perturbed intermittently throughout the different reactor cycles, which consist of a start-up phase (∼12 h), operational phase (20 to 30 days), and shutdown phase (<1 h) and directly affected conductivity, pH, and temperature. We focused on the three most prevalent taxa in this system: one that was ubiquitous and abundant, and two that were sporadically abundant (i.e., conditionally rare [30]). We used genome-centric metagenomics to infer phylogeny, genome properties, and physiological traits (i.e., growth rate and optimal growth temperature), as well as comparative genomics to identify functional adaptations and positively selected genes (PSGs). We isolated a representative of the most prevalent bacterioplankton taxon and phenotypically characterized it to help confirm its adaptation to highly nutrient-depleted conditions, as inferred from *in situ* population dynamics and metagenomic analyses.

## RESULTS AND DISCUSSION

### Oligotrophic cooling water contains a highly uneven bacterial community without significant intertaxon covariation.

**FIG 1:**
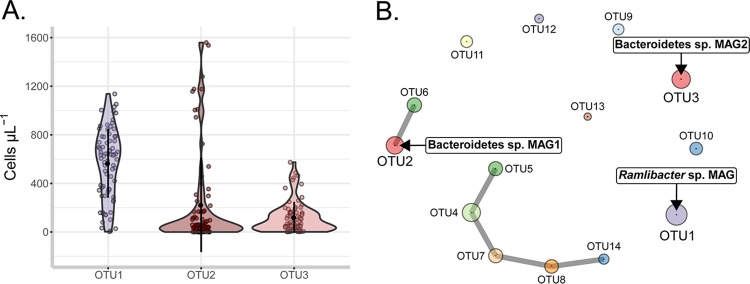
(A) Absolute abundances of the *Ramlibacter* sp. OTU (OTU1) and the two abundant *Bacteroidetes* sp. OTUs (OTU2 and OTU3) expressed in number of cells milliliter^−1^ in two sampling surveys (*n* = 77) of the cooling water system previously described by Props et al. (31). Abundances were estimated by multiplying relative abundances from amplicon sequencing of the V3-V4 16S rRNA gene region with the cell counts estimated through flow cytometry. (B) Association network of the most prevalent OTUs in the amplicon survey data using the sparse inverse covariance estimation method (SPIEC-EASI) (95). The node radius is proportional to the median of centered log transformed abundances. The metagenome-assembled genomes (MAGs) associated with specific OTUs are annotated next to the OTU nodes (text boxes).

**(i) Nutrient and population dynamics.** We previously surveyed at high resolution the microbial community inhabiting the oligotrophic cooling water reservoir (2,500 m³) that was characterized by low conductivity (3.4 ± 1.4 µS cm^−1^), pH (4.5 ± 0.2), NO_3_^−^-N (62 ± 50 µg liter^−1^), PO_4_^3−^-P (limit of detection < 1 µg liter^−1^), and total organic carbon (TOC) (2.36 ± 0.08 mg liter^−1^) (31). The system was strongly depleted of orthophosphate (NO_3_^−^-N/PO_4_^3−^-P molar ratio > 96), for which the measured concentration was likely an overestimation of the bioavailable phosphate fraction (32). A longitudinal survey of the microbial community of the system (16S rRNA gene amplicon sequencing of V3-V4 region) during its 30-day operational cycles revealed a single betaproteobacterium and two conditionally rare *Bacteroidetes* taxa (operational taxonomic units [OTUs]) to account for more than 90% of the community (31). The betaproteobacterium was classified as a member of the betI-A freshwater clade (Fig. 1A, OTU1), which is comprised of the ubiquitous freshwater bacterioplankton genus *Limnohabitans*, as well as the uncultured GKS16 lineage (33, 34). The *Bacteroidetes* OTUs were classified into the bacI-A freshwater clade (*Chitinophagaceae* family) (Fig. 1A, OTU2 and OTU3). The abundant OTUs did not exhibit strong ecological interactions with other OTUs throughout the 30-day reactor operation as inferred from the association network (Fig. 1B), and their abundances were not strongly correlated with temperature, pH, or nutrient levels (see Fig. S1 posted at https://doi.org/10.6084/m9.figshare.7577636).

**FIG 2:**
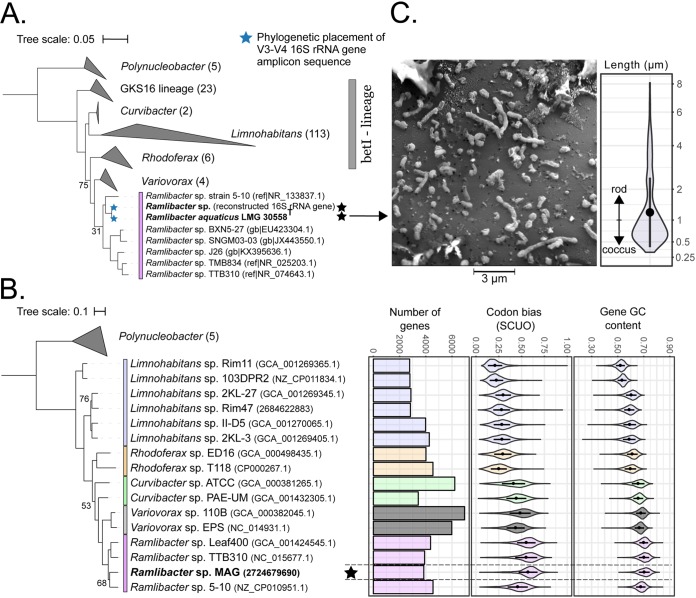
(A) 16S rRNA gene tree of abundant bacterioplankton groups within the putative betI lineage. The tree was rooted in Escherichia vulneris (GenBank accession no. AF530476.1) and Enterobacter cancerogenus (emb|Z960781). (B) Phylogenomic tree based on a concatenated codon alignment of 37 conserved gene families of representative isolate genomes with Chitinophaga niabensis as outgroup (IMG id 2636416022). The number of genes, codon usage bias (SCUO), and gene GC content for each genome are indicated by a horizontal bar or violin plot. Genomes are colored by genus. For both trees, only bootstrap values of <80 are reported. (C) Scanning electron microscopy (SEM) (10 keV) image of a late-stationary-phase *R. aquaticus* LMG 30558^T^ culture isolated from the ecosystem. Length distribution (*n* = 166) of *R. aquaticus* was calculated based on five different SEM images using ImageJ. Additional SEM images are available at https://doi.org/10.6084/m9.figshare.7472204.

**(ii) Population genome reconstruction and taxonomic classification.** To investigate the genomic adaptations in this nutrient-depleted environment, we performed whole-genome shotgun sequencing to reconstruct the population genomes of the three prevalent OTUs. Metagenome-assembled genomes (MAGs) were estimated to be complete for all three taxa (Table 1). The betI-A MAG was classified as a *Ramlibacter* species after classification of its constituent contigs using both the *Phylosift* and *Centrifuge* classification tools. This classification was further confirmed through phylogenetic placement of the reconstructed full-length 16S rRNA gene sequence into a 16S rRNA gene reference tree consisting of betI taxa and other freshwater clades (Fig. 2A). As *Ramlibacter* species have been observed only in (extreme) terrestrial environments (35–37), we constructed a phylogenomic tree based on a concatenated codon alignment of 37 conserved genes found in publicly available *Ramlibacter* genomes and a representative set of genomes belonging to the freshwater taxonomic groups used in the 16S rRNA gene tree (Fig. 2B). The *Ramlibacter* sp. MAG was part of the monophyletic *Ramlibacter* clade, and its GC content (70.6%), codon usage bias (SCUO = 0.57 ± 0.10), and genome size (3.95 Mbp) closely matched these of other *Ramlibacter* genomes (Fig. 2B). As the *Ramlibacter* clade was not previously identified in freshwater environments, it has not been considered as part of the betI lineage. Our analysis showed that it forms a separate clade from the betI lineage and that its closest betI relatives are possibly in the betI-B clade (*Rhodoferax* genus). Interestingly, none of these populations were found in the groundwater feed of the system at the time of sampling, indicating that they were in low abundance or that they originated from alternative sources, such as the different groundwater sources that are intermittently used by the system. Further analysis of 55 publicly available freshwater metagenomic data sets revealed that no close relatives to these populations are present in currently sampled lakes and rivers worldwide (https://doi.org/10.6084/m9.figshare.7472978.v1). The presence of these populations remains enigmatic, as they must possess specific adaptations to this unique freshwater environment.

**TABLE 1:** Assembly statistics and genome properties for the three metagenome-assembled genomes (MAGs) in this study

Assembly statistic or genome property	*Ramlibacter* sp. MAG	*Bacteroidetes* sp. MAG1	*Bacteroidetes* sp. MAG2
Total length (Mbp)	3.95	3.43	3.72
No. of contigs	109	26	140
*N*_50_ (bp)	54,423	253,522	45,757
Estimated % completeness (% redundancy)	100 (1.4)	100 (0.0)	100 (1.4)
GC content (%)	70.6	45.3	34.5
Coding density (%)	92.4	91.9	91.7
Mean coverage range across samples	8.61–33.4	0.24–23.5	1.77–6.69
No. of genes	3,847	3,159	3,361
% KO annotated genes	53.8	39.2	40.6
% COG annotated genes	73.6	56.3	57
% Pfam annotated genes	85.9	78.5	77.3
No. of putative HGT genes	145	58	72
No. of paralog genes	926	433	499
No. of σ-factor homologs	16	25	20
Predicted growth rate^*a*^ (h^−1^)	0.19 ± 0.02	0.18 ± 0.02	0.16 ± 0.02
Predicted OGT^*a*^ (°C)	29	16	18
IMG taxon id	2724679690	2724679698	2724679691

a Growth rate and optimal growth temperature (OGT) predicted using Growthpred (v1.07).

**(iii) Isolation of *Ramlibacter* strain LMG 30558^T^.** We isolated a representative of the *Ramlibacter* MAG in order to compare its physiological and growth behavior with those reported for oligotrophic and streamlined taxa (see the data posted at https://doi.org/10.6084/m9.figshare.7577636). The 16S rRNA gene sequence was >99% similar to the metagenomic reconstructed 16S rRNA gene sequence, and the phylogenetic placement of the V3-V4 16S rRNA gene amplicon sequence had equal likelihood on both the reconstructed and isolate sequence terminal branches (Fig. 2B). The most closely related formally classified organism was Ramlibacter solisilvae (with LMG 30470^T^ as type strain) with a 16S rRNA gene identity of 96.7% between these two strains, demonstrating that this species represents a novel and the first observed aquatic species of the *Ramlibacter* genus (38). This was confirmed by the low average nucleotide identity (80.1%, <50% coverage, blast method) between the two genome sequences. As this organism was also phenotypically distinguishable from its nearest neighbor species, we propose to formally classify it as Ramlibacter aquaticus sp. nov. with strain LMG 30558 as the type strain. For detailed characterization, see the phenotypic description section in Materials and Methods. The assembled genome of R. aquaticus LMG 30558^T^ was 100% identical (ANIb and ANIm methods) to that of the *Ramlibacter* sp. MAG, but its assembly was of lesser quality. Therefore, we based our inference of the *R. aquaticus* LMG 30558^T^ genome on the *Ramlibacter* sp. MAG throughout the following analyses, but we kept the MAG labeling consistent across the figures to clearly indicate that this represents a metagenome-assembled genome.

### Prevalent populations lack streamlining characteristics.

Although specific genome streamlining properties have been detected in other bacterioplankton taxa (i.e., characteristic genome size, coding density, GC content, and number of paralog genes and sigma factors [7, 9, 10, 23, 25, 39]), we found no conclusive evidence of genome streamlining in the three prevalent taxa in our ecosystem (Table 1). All three taxa had genomes that were average in size for bacteria (∼3.5 to 4.0 Mbp) but maintained a relatively high coding density between 91.7% (*Bacteroidetes* sp. MAG1) and 92.4% (*R. aquaticus* LMG 30558^T^). Intermediate streamlined genomes such as *Polynucleobacter* sp. have similar coding densities (∼92%) but smaller genomes (∼2 Mbp) (25) while extremely streamlined genomes, such as *Pelagibacter* and freshwater *Actinobacteria* have even smaller genomes (<1.5 Mbp) and higher coding density (>95%) (9, 10). Typical coding densities for bacteria range from 85% to 90% (10). Interestingly, and similar to other *Ramlibacter* isolates (40), the *R. aquaticus* LMG 30558^T^ isolate could modulate its cell length from a typical “streamlined” cell length of <1 µm (41) up to 8-µm elongated rods (Fig. 2C). In comparison, for the few cultivated streamlined taxa, a fixed cell size (i.e., ≤1 µm) and morphology have been reported (10, 23).

The average (gene) GC content matched those of most streamlined genomes (i.e., <50%) for the *Bacteroidetes* MAGs (34.5% and 45.3%), but not for *R. aquaticus* LMG 30558^T^ which had one of the highest reported for the *Betaproteobacteria* (i.e., 70.6%) (36). The number of paralogous genes was the highest in *R. aquaticus* LMG 30558^T^ comprising approximately 24% of all genes, suggesting considerable gene expansion through either horizontal gene transfer (42) or gene duplication events (43). The number of sigma factor genes was lowest for *R. aquaticus* LMG 30558^T^ in the ecosystem studied (i.e., 16 genes), but it was comparable to those of the terrestrial *Ramlibacter* genomes (14 to 21 genes; see Fig. S2 at https://doi.org/10.6084/m9.figshare.7577636). While higher than for nutrient-driven streamlined genomes such as those of *Pelagibacter* sp. (≤10), the number of sigma factors was lower than predicted (∼30) based on existing correlations between genome size and genome properties (10). Overall, the genome properties of *R. aquaticus* LMG 30558^T^ were in line with those of other *Ramlibacter* genomes.

### Genes under positive selection.

As *R. aquaticus* LMG 30558^T^ was the dominant population, we focused on discovering genes that have been under positive selection and thus may have facilitated adaptation to a nutrient-depleted aquatic environment. Positively selected genes (PSGs) were searched for in the *R. aquaticus* LMG 30558^T^ branch and in the last common ancestor (LCA) of all available *Ramlibacter* sp. genomes.

**FIG 3:**
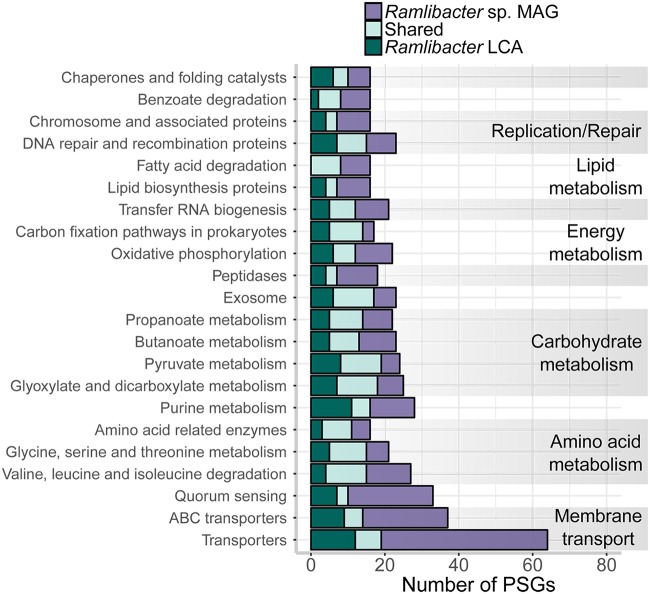
KEGG annotation of homologous genes under positive selection in only *Ramlibacter* sp. MAG (*R. aquaticus* LMG 30558^T^), in only the *Ramlibacter* LCA, and shared between the *Ramlibacter* sp. MAG (*R. aquaticus* LMG 30558^T^) and the *Ramlibacter* sp. LCA (shared group). Genes that were involved in multiple categories were counted in each category. Only KEGG subsystem categories containing more than 15 PSGs are shown.

**(i) Overview of genome-wide PSG detection.** In relation to other genomes included in the tree (Fig. 2B), we observed 485 PSGs (12.6% of all genes) spread across a wide range of metabolic pathways in *R. aquaticus* LMG 30558^T^ (*P* < 0.05 and FDR < 0.05; Fig. 3). There were 291 PSGs in the *Ramlibacter* LCA, 150 of which were orthologs with the PSGs in *R. aquaticus* LMG 30558^T^. There were no KEGG subsystem categories enriched in the positively selected gene pool (*P* > 0.05). However, across these functional categories, we identified a large number of PSGs that were involved in branched-chain amino acid (BCAA) transport (15 genes) and metabolism (12 genes). These specialized transporters were all high-affinity ABC-type transporters specialized in the uptake of BCAAs at low concentrations: they are abundant in all *Ramlibacter* genomes (36) and are also present in streamlined genomes (9, 39). BCAAs represent nitrogen-rich organic carbon sources (44), but they are also important precursors for the synthesis of DSF-type signal molecules in Gram-negative bacteria, which can regulate motility, biofilm formation, extracellular enzyme synthesis, and extracellular polymeric substance production (45). *R. aquaticus* LMG 30558^T^ possibly exhibits this recently discovered cell-to-cell communication pathway, as four putative copies of the key DSF biosynthesis enzyme enoyl-CoA hydratase were found, with one being under positive selection.

Across the lipid and carbohydrate metabolism categories, multiple genes involved in the metabolism of polyhydroxyalkanoate (PHA), the sole energy and carbon reserve biopolymer of *R. aquaticus* LMG 30558^T^, were under selection. Nearly all genes necessary for the production of PHA building blocks from the fatty acid β-oxidation pathway (i.e., hydroxyacyl-CoA), as well as the PHA synthase and PHA depolymerase were under positive selection. As such, the ability of *R. aquaticus* LMG 30558^T^ to store carbon and energy as PHA appears to have been an important adaptation to its overall nutrient-depleted environment.

**FIG 4:**
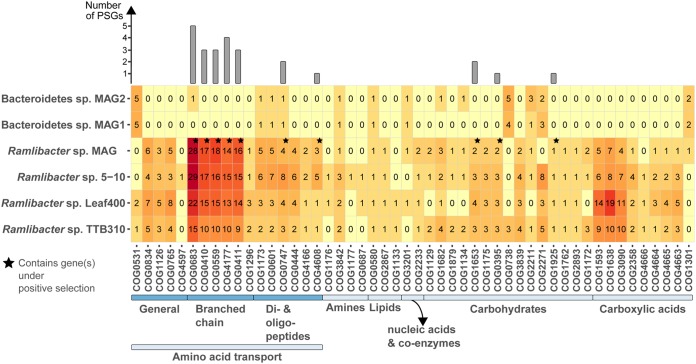
Frequencies of dissolved organic carbon (DOC) transporter genes in the *Ramlibacter* sp. MAG (*R. aquaticus* LMG 30558^T^), publicly available *Ramlibacter* terrestrial isolate genomes, and the two *Bacteroidetes* MAGs reconstructed in this study. Each column represents a single COG.

**(ii) Dissolved organic carbon scavenging.** As carbohydrate as well as amino acid transport and metabolism represented a large fraction of the gene content under positive selection in *R. aquaticus* LMG 30558^T^ (i.e., 21%), we investigated the overall diversity in dissolved organic carbon (DOC) uptake mechanisms. For this analysis, we relied on a curated list of DOC transporter genes relevant for aquatic microbial taxa (Fig. 4) (24, 46). All *Ramlibacter* sp. had a large gene arsenal dedicated to the transport of a wide range of DOC classes, but in particular branched-chain amino acids and di- and oligopeptides. In contrast, the *Bacteroidetes* MAGs had primarily general and carbohydrate-focused transport systems coupled with a high abundance and diversity in glycoside hydrolase (GH) gene classes (see Fig. S3 posted at https://doi.org/10.6084/m9.figshare.7577636). The most abundant GH classes were targeting complex cellulosic biomass and fucose-containing polysaccharides (i.e., GH3, GH29, and GH74). The ability of the *Bacteroidetes* taxa to be competitive primarily at elevated concentrations of complex DOC, similar to other members of the bacI-A clade, may explain why they are conditionally rare in our system (33).

**FIG 5:**
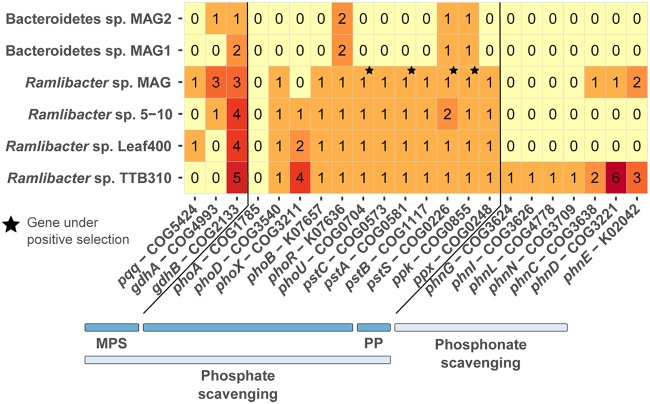
Frequencies of phosphorus scavenging genes in the *Ramlibacter* sp. MAG (*R. aquaticus* LMG 30558^T^), publicly available *Ramlibacter* terrestrial isolate genomes, and the two *Bacteroidetes* MAGs reconstructed in this study. MPS, mineral phosphate solubilization; PP, polyphosphate.

**(iii) Phosphorus scavenging.** As the environment had an extremely low orthophosphate concentration (see Table S1 posted at https://doi.org/10.6084/m9.figshare.7577636), we evaluated whether there was selection on, or enrichment of, phosphate-dedicated scavenging genes (Fig. 5). *R. aquaticus* LMG 30558^T^ had the pyrroloquinoline quinone cofactor-encoding gene (*pqq*), which coupled with dehydrogenase activity (*gdhAB*) constitutes a known mechanism of phosphate scavenging through mineral phosphate solubilization (MPS) (47). All other *Ramlibacter* spp. lacked the key *pqq* cofactor for MPS. The *Bacteroidetes* MAGs had no means for phosphate scavenging apart from a single high-affinity ABC-type phosphate transporter gene (e.g., *pstB*). There was only one alkaline phosphatase gene (*phoD*) detected in *R. aquaticus* LMG 30558^T^ whereas multiple (multicopy) genes (*phoX*) were present in the other *Ramlibacter* spp. (48). The capacity of *R. aquaticus* LMG 30558^T^ and the *Bacteroidetes* MAGs to scavenge phosphate from organic components was thus limited. In *R. aquaticus* LMG 30558^T^, the polyphosphate kinase (*ppk*) gene, two high-affinity inorganic phosphate transporter (*pstAS*) genes, and one phosphate uptake regulator gene (*phoU*) were under positive selection. All *Ramlibacter* sp. genomes had the genes necessary for storing phosphorus in polyphosphate granules (i.e., *ppk* and *ppx* genes). Although *R. aquaticus* had the apparatus for the transport of organophosphonates (*phnCDE*) (49), a potentially abundant phosphorus source in ultraoligotrophic environments (50), it lacked multiple genes required for their utilization (e.g., *phnGILN*). The positive selection on both DOC and phosphorus scavenging pathways are in accordance with recent work highlighting the coupling of carbon and phosphorous cycles in freshwater microbial communities (51).

### *Ramlibacter* sp. MAG accessory genome.

The adaptation of *R. aquaticus* LMG 30558^T^ to its environment may have been facilitated by not only positive selection on gene sequence but also selection for the preservation of (lineage-specific) gene expansions. We determined the core and accessory genome of *R. aquaticus* LMG 30558^T^ and tested for the enrichment of KEGG subsystem categories by means of a *Ramlibacter* pangenome analysis (see Fig. S4 posted at https://doi.org/10.6084/m9.figshare.7577636).

The accessory genomes of *R. aquaticus* LMG 30558^T^ (585 genes, 15% of genome) and the other *Ramlibacter* sp. were primarily occupied by membrane transport and signal transduction genes. The accessory genome of *R. aquaticus* LMG 30558^T^ was functionally enriched (*P* < 0.001) in chemotaxis, cell motility, transport, and two-component transduction pathways (see Fig. S5 posted at https://doi.org/10.6084/m9.figshare.7577636). These subsystem categories comprised 26% of the accessory genome and 89% of the annotated fraction. This was substantially higher than the enriched functional groups of the other *Ramlibacter* genomes which varied between 4% and 49% of the annotated fraction (see Fig. S5 at the URL mentioned above). The enrichment of these specific subsystem categories are likely adaptations of *R. aquaticus* LMG 30558^T^ to its environment as recent mathematical evolutionary models have shown that gene additions are, on average, adaptive processes, and not neutral (52). *R. aquaticus* LMG 30558^T^ had the most diverse chemotaxis and flagellar gene inventory, including two aerotaxis receptor genes (see Fig. S6 at the URL mentioned above). Phosphorus-driven chemotaxis and cell size plasticity (1 to >5 µm) similar to what we observed in *R. aquaticus* LMG 30558^T^ have been shown to be adaptations to phosphorus starvation of *Thallasospira* spp. (genome size > 4 Mbp), which are often found in ultraoligotrophic marine environments (53). We hypothesize that *R. aquaticus* LMG 30558^T^ may elicit this behavior as well; its population genome was annotated with a complete chemotaxis pathway and many phosphorus scavenging mechanisms, while its cells display substantial morphological plasticity.

### Culture- and genome-based evaluation of growth characteristics of *R. aquaticus* LMG 30558^T^.

**FIG 6:**
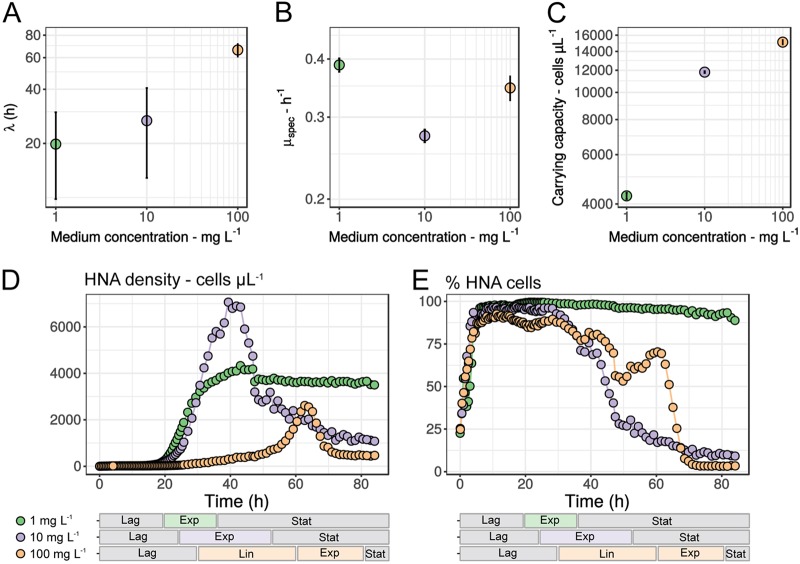
Growth kinetics of *R. aquaticus* LMG 30558^T^ under different initial medium concentrations (R2A broth). (A) Length of lag phase (spline fit). (B) Specific growth rate (logistic model). The specific growth rate estimation at 100 mg liter^−1^ was less reliable due to the presence of both a linear and an exponential growth phase, which resulted in a less optimal fit of the logistic model. (C) Carrying capacity (spline fit). (D) Cell density dynamics of the putative high-productivity cell population (HNA) during batch growth. (E) Relative abundance dynamics of the putative high-productivity cell population (HNA) during batch growth. Cell density dynamics of total cells and LNA population are provided in Fig. S9 posted at https://doi.org/10.6084/m9.figshare.7577636. Horizontal stacked bar charts positioned below the graphs in panels D and E indicate the observed growth phases for each medium concentration (Lag, lag growth phase; Exp, exponential growth phase; Lin, linear growth phase; Stat, stationary growth phase).

Based on current bacterial lifestyle classification systems, taxa with the genome profile of *R. aquaticus* LMG 30558^T^ are predicted to have a copiotrophic lifestyle that is suited to quickly exploit nutrient pulses (39, 54). However, given the low nutrient levels of our study system, we hypothesized that *R. aquaticus* LMG 30558^T^ would need to exhibit high growth rates even at low nutrient concentrations in order to be competitive and maintain its prevalence. Therefore, we assessed the growth kinetics of *R. aquaticus* LMG 30558^T^ at different nutrient concentrations (Fig. 6A to C) and compared these with genome-based predictions of specific growth rates and optimal growth temperature (Table 1). As hypothesized, *R. aquaticus* LMG 30558^T^ exhibited optimal growth at the lowest concentration of 1 mg liter^−1^ R2A with a lag phase of 20 h (95% CI, 10 to 30 h), a specific growth rate of 0.39 ± 0.01 h^−1^, and a generation time of 1.8 h (95% CI, 1.7 to 1.9 h). Its specific growth rate was double the growth rate predicted from its genomic properties (Table 1), 20 times higher than those recorded for *Pelagibacter* spp. (55) and more than double than those of many *Polynucleobacter* isolates (56). Furthermore, counter to the expected copiotrophic behavior (i.e., growth rate is conditional on nutrient concentration), increasing the medium concentration over 2 orders of magnitude did not lead to improved growth kinetics. Instead, the growth rate and lag phase length were negatively impacted by the larger resource availability, while the carrying capacity appeared to reach its maximum at the highest concentration. It is important to note that as *R. aquaticus* LMG 30558^T^ was isolated from a low-conductivity environment, the (hyper)osmotic stress at higher medium concentrations may also impact its growth kinetics. Further research is necessary to specifically reveal its physiological response to changing osmolarity, as its genome contains several key genes related to osmoregulation (i.e., OmpR family two-component system and ABC-type osmoprotectant transport). Interestingly, *R. aquaticus* LMG 30558^T^ grew optimally at 28°C throughout our subcultivations (see phenotypic description section below), which could also be predicted from its genomic properties (i.e., optimal growth temperature [OGT] of 29°C; Table 1), and was close to the average temperature prevailing in the ecosystem during our monitoring campaign (i.e., 27°C). Meanwhile, the *Bacteroidetes* MAGs had lower OGTs (i.e., 16 to 18°C), again indicating a lower fitness in the studied ecosystem.

To further demonstrate that *R. aquaticus* LMG 30558^T^ was adapted to a low-nutrient environment, we tracked the abundance of the high-nucleic-acid-content (HNA) bacteria throughout the growth phases. This is a common, but debated, proxy for the bacterial fraction with the highest (secondary) productivity in aquatic communities, as it typically exhibits the highest amino acid assimilation rates (57, 58) (Fig. 6D and E; see Fig. S7 and S8 posted at https://doi.org/10.6084/m9.figshare.7577636). Only at the lowest medium concentration (1 mg liter^−1^ R2A) did the HNA cell density remain consistently high after the exponential phase, while for the other growth conditions the HNA cell density quickly decreased to minimal levels (Fig. 6D). In terms of relative abundances, the 1 mg liter^−1^ R2A growth condition maintained near-constant percentage of HNA cells, while the other growth conditions experienced a sharp decrease in percent HNA cells after approximately 30 h of incubation (Fig. 6E). This sharp transition cooccurred with the exponential and/or linear growth phases and resulted in the majority of growth to take place in the low-nucleic-acid-content (LNA) population. The high percentage of HNA cells in the 1 mg liter^−1^ R2A growth condition is in accordance with the observed percentage of HNA cells in the freshwater where *R. aquaticus* LMG 30558^T^ was dominant (86% ± 5%) (see Table S2 posted at the above URL). The relationship between HNA abundance and productivity has so far been reported only for microbial communities and not for individual taxa (59, 60), as it is still unclear whether, and by which physiological mechanism taxa can migrate between HNA and LNA fractions (57, 61). Here we showed that *R. aquaticus* LMG 30558^T^ can actively switch between these nucleic acid populations and that the majority of replication in this population occurs solely during the exponential growth phase. While we cannot specify the exact mechanism how this switch occurs, it is likely a combination of its pleomorphic behavior (i.e., larger cells contain more nucleic acids [Fig. 2C]), chromosome copy number variation (61, 62), transcriptional activity, and a modulation of its abundant membrane transport processes (dye permeability), possibly in response to nutrient stress (both limitation and excess).

Overall, the observed and predicted growth kinetics of *R. aquaticus* LMG 30558^T^ are not in line with those used in the binary classification of streamlined oligotrophs and larger-genome copiotrophs, but fit more into the “fast, reduced responders” category proposed by Livermore et al. (54). Similar to *R. aquaticus* LMG 30558^T^, this category is characterized by genomes of intermediate size (up to ∼3.9 Mbp), limited diversity in carbon substrate utilization, and a high investment in environmental sensing capabilities (54). However, unlike the “fast reduced responders” category, *R. aquaticus* LMG 30558^T^ maintains a high amino acid assimilation rate (percent HNA cells as proxy) at low-nutrient conditions and does not respond positively to log fold increases in nutrient concentrations. The sum of these physiological and genome-predicted features indicate that the *R. aquaticus* LMG 30558^T^ population may be adapted to the rapid exploitation of low-nutrient and carbon pulse patches released in the cooling water system. The estimated assimilable organic carbon (AOC) (conversion factor of 10^7^ cells µg C^−1^ [63]) released during the start-up of the reactor (i.e., 92 to 146 µg C liter^−1^, calculated from the cell density dynamics from Props et al. [31]) certainly agrees with the predicted adaptation to low nutrient or carbon pulses. We therefore argue the importance of evaluating the growth kinetics (i.e., “responsiveness”) of bacterial taxa under both xenic (31, 64) and axenic conditions across a range of nutrient concentrations for advancing future theory development.

### Conclusions.

Our results suggest that *R. aquaticus* LMG 30558^T^, the dominant population in our system, has evolved a hybrid strategy to survive in a nutrient-depleted environment that experiences strong dynamics in temperature and conductivity. First, its coding density has been optimized while minimizing gene loss and the number of regulatory elements. Second, evolutionary forces have optimized its nutrient scavenging potential through positive selection on key genes in nutrient and carbon scavenging, and expanding its relevant genetic repertoire. Third, it can potentially increase its surface-to-volume ratio through morphological plasticity and exhibits optimal growth kinetics at low nutrient concentrations. *R. aquaticus* LMG 30558^T^ is thus a prime example of an aquatic taxon that does not show distinct ecological interactions with other taxa and cannot easily be placed in the current dichotomous streamlining and oligo/copiotroph framework. Our results highlight that genome streamlining is not the sole adaptive strategy under nutrient-depleted conditions (28) and adds to other reports that propose more diverse classification systems for genomic adaptations (54).

## MATERIALS AND METHODS

### Cooling water system.

The cooling water ecosystem was part of a discontinuously operated nuclear research reactor (50 to 70 MW). This system was temporarily challenged with step pulses in conductivity (from 1 to 7 µS cm^−1^) and temperature (from 15 to 30°C) during start-up and subsequent operation. After 20 days of operation, the reactor was shut down for at least one month, thereby returning conductivity and temperature to their initial state (i.e., ∼1 µS cm^−1^ and 10 to 15°C). The microbial community studied here was situated in the central reservoir (2,500 m³, situated directly below the cooling tower cascade) and was continuously cycled through a series of ion exchangers to maintain its low conductivity. During reactor operation, the cooling water was fed to the secondary cooling circuit at a flow rate of 4,000 m³ h^−1^ and circulated back to the central reservoir over a cooling tower cascade. Evaporated water was continuously replenished by a deionized and iron-depleted (<2 µS cm^−1^) groundwater feed at a flow rate of approximately 70 m³ h^−1^. The community was sampled from the central reservoir throughout two 25- to 30-day reactor cycles with an average sampling frequency of two times per day.

### Whole-genome shotgun sequencing.

Four samples previously analyzed as part of a 16S rRNA gene amplicon sequencing survey of the secondary cooling water microbial community of a nuclear reactor were chosen for whole-genome shotgun sequencing (31). The samples were taken during the operational phase of the reactor in two different reactor cycles and were chosen to maximize differential coverage of the three predominant populations across the samples. The DNA was extracted according to a previously published protocol (65). Libraries were prepared using the Nextera XT kit (Illumina Inc.) and sequenced on a 2 × 250-bp paired-end Illumina MiSeq run using the MiSeq V2 kit. Approximately one million reads were generated for each sample with an insert size of 420 bp (see Table S1 posted at https://doi.org/10.6084/m9.figshare.7577636). Raw reads were evaluated using FastQC. *Sickle* (v1.33.6) (https://github.com/najoshi/sickle) was used for removing erroneous and low-quality reads from the raw data. *Scythe* (v0.993) (https://github.com/vsbuffalo/scythe) was used for removing adapter contaminant sequences. The reads were dereplicated using an in-house *perl* script and interleaved into a single sequence file for subsequent coassembly (66).

### 16S rRNA gene reconstruction.

We reconstructed full-length 16S rRNA gene sequences from quality-trimmed reads using *EMIRGE* (v0.60.3) (67). *EMIRGE* was run using both the nonredundant 97% clustered Silva database (v123) (68) and the freshwater database (FWDB) (69) as references. *EMIRGE* was run with the quality-trimmed reads and with the insert size and standard deviation parameters e set at 500 to maximize read mapping. We ran two *EMIRGE* analyses, using merge option -j at values of 0.97 and 1.0 and merged the reconstructed sequences into a single sequence file. *EMIRGE* reconstructed sequences with a normalized prior abundance of <5% were removed, and sequences were ordered from high to low abundance before clustering at 97% similarity by UCLUST. Classification of the sequences was performed using the *TaxAss* pipeline which combines both the Silva v123 database and the FWDB (69). FWDB classification was favored over the Silva classification if the length-corrected identity to a FWDB sequence was >95%.

### Genome reconstruction.

The interleaved reads of all four samples were coassembled into contigs using *IDBA-UD* (v1.1.3) with kmer lengths varying from 41 to 101 in steps of 10 (70). Contigs were taxonomically classified at the order level by a diamond search against the nonredundant NCBI protein database using *DESMAN* scripts (71) (*classify_contigNR.pl* with MIN_FRACTION = 0.1), after which the classification output files were formatted into an annotation file compatible with the *Vizbin* binning tool. Initial metagenome-assembled genomes (MAGs) were retrieved through a manual binning strategy in *Vizbin* (v0.9, default settings, minimum contig length of 1,000 bp) (72). Quality-trimmed reads were mapped to the coassembly using *bwa-mem* (v0.7.8) on default settings (73). *Samtools* (v1.3.1) was used to convert, sort, and index the sam files (74). The *Anvi'o* platform (v2.3.0) was then used to manually refine the MAGs identified through *Vizbin* by evaluating differential coverage patterns across the samples. Completeness and redundancy estimates of all MAGs were estimated through the bacterial and archaeal marker gene databases in *Anvi'o* (75) as well as the lineage-specific marker sets in *CheckM* (v1.07) (76). The refined MAGs were submitted for gene calling and annotation to the Joint Genome Institute’s Integrated Microbial Genomes isolate annotation pipeline (77). Codon usage bias was inferred from the synonymous codon usage order (SCUO) (*CodonO* software), which is a metric comparable across genomes (78). For each MAG and selected publicly available freshwater genomes, the minimal generation time (MGT) and optimal growth temperature were predicted based on “growth-imprinted” genome features by means of the *Growthpred* (v1.07) software (15). The predicted specific growth rates (µ_spec_) were then calculated by the following formula: $\mu_{\text{spec}}=\ln2/\text{MGT}\text{.}$

The presence of closely related populations in publicly available data sets used by Neuenschwander et al. (9) was assessed by blasting one million reads of each data set to the metagenomic assembly of the cooling water and assessing the read alignment across the contigs of the MAGs. Samples that had reads mapping with high identity (≥95%) and that were homogenously distributed across the contigs of the MAGs were considered to contain a closely related population to the MAG(s).

### Phylogenetic and phylogenomic tree construction.

A 16S rRNA gene phylogenetic tree was constructed using a set of publicly available betI clade, *Ramlibacter* genus, and other related lineage sequences. Sequences were aligned using the SINA aligner (79). The tree was constructed with *Fasttree* (v2.1.9) using the GTR+CAT evolutionary model (80). Phylogenetic placement of the V3-V4 OTU consensus sequence on the reference tree was conducted using *PhyloAssigner* (v6.166) (81). The phylogenomic tree was constructed based on the codon alignment of a set of 37 conserved marker genes through *Phylosift* (82). The tree was generated from the codon alignment by means of *RAxML* (v8.2.8) with the GTRGAMMA model and 100 bootstraps (83). Both trees were visualized and annotated in *iTOL* (84) and exported and further annotated in *Inkscape* (v0.91).

### Inference on positively selected genes.

We used *PosiGene* to infer positively selected genes (PSGs) in the *Ramlibacter* MAG (85). The *Ramlibacter* MAG was used as the anchor, reference, and target species to ensure that only genes present in this genome were tested for positive selection. The selected genomes previously used in the phylogenomic tree were used to create ortholog groups, make the phylogenomic tree, and create high-quality codon alignments (-nshbr flag) to perform the branch site test for positive selection. We performed the same analysis on the *Ramlibacter* last common ancestor (LCA) branch. Genes were considered PSGs if the branch-wide test resulted in a false discovery rate (FDR) of <0.05 and an adjusted *P* value of <0.05 and had a ratio of substitution rates at nonsynonymous and synonymous sites $(\frac{d_{N}}{d_{S}}\;-\;\omega ) < 30.$

### Pangenome analysis.

We applied the pangenome analysis workflow available in *Anvi’o* (v2.3.0) to all *Ramlibacter* genomes (75). Gene calling was performed with *prodigal* (v2.6.2), amino acid sequences were aligned with *muscle* (v3.8.31) (86), amino acid similarities were calculated with *blastp* (v2.2.29), and sequences were clustered with *mcl* (v14-137) (87). Accessory genomes were manually binned in *Anvi’o* (see Fig. S2 posted at https://doi.org/10.6084/m9.figshare.7577636). As there were no IMG annotation projects available for the other *Ramlibacter* genomes, the gene calls from *Anvi’o* were exported and annotated with KEGG orthology identifiers using the BlastKOALA web server to facilitate direct comparison between genomes (88). Functional enrichment analysis was conducted with the hypergeometric testing available in the clusterProfiler package (v3.6.0) (89). *P* values were adjusted for multiple testing with the Benjamini-Hochberg correction.

### Isolation and growth kinetics.

A fresh 2-liter sample from the cooling water was collected on 23 October 2017 when the reactor was not operational (water temperature between 10 and 15°C and conductivity of <7 µS cm^−1^) and stored at 4°C. Initial enrichments were performed by means of dilution to extinction in 96-well multiwell plates at three different concentrations (0.1 mg liter^−1^, 1 mg liter^−1^, and 10 mg liter^−1^) of R2A broth (Lab M, Heywood, United Kingdom). Plates were incubated in the dark at 28°C for 2 weeks. Wells with growth (>10^5^ cells ml^−1^) were transferred to fresh medium and incubated for 2 weeks under identical conditions. The actively growing cultures were filtered on 0.2-µm filters and their DNA was extracted using a previously published protocol (65). The full-length 16S rRNA gene was amplified and Sanger sequenced (primers 24F and 1492R). A *Ramlibacter* strain (formally classified as *Ramlibacter aquaticus* LMG 30558^T^) was successfully isolated at 1 mg liter^−1^ R2A and kept in active cultivation. Growth was evaluated by means of flow cytometry as described previously (65). Scanning electron microscopy (SEM) images were taken as described in the information posted at https://doi.org/10.6084/m9.figshare.7577636. A phenotypic analysis of *R. aquaticus* LMG 30558^T^ and of Ramlibacter solisilvae LMG 30470^T^, its nearest phylogenetic neighbor species, was performed as described before (90). Inoculated plates were incubated for 10 days before test results were interpreted.

Growth curves of the *R. aquaticus* LMG 30558^T^ were acquired by means of online flow cytometry as previously demonstrated using the OnCyt sampling and staining module (OnCyt, Zürich, Switzerland) (91, 92). *R. aquaticus* LMG 30558^T^ was grown for 7 days in 1 mg liter^−1^ R2A after which it was used to inoculate three batch reactors (0.5-liter Schott glass bottles) at approximately 10^4^ cells ml^−1^. The three reactors contained 200 ml of 1 mg liter^−1^ R2A, 10 mg liter^−1^ R2A, and 100 mg liter^−1^ R2A and were incubated for 4 days in a transparent incubator at 28°C. Samples were taken approximately every 20 min (first 69 samples) or every 40 min (subsequent samples), stained with 1,000-fold diluted SYBR Green I (TRIS buffer at pH 8.2), and incubated for approximately 20 min at 37°C prior to analysis. For each sample, 103.8 ± 2.6 µl was sampled for the first 69 measurements, after which 32.6 ± 0.3 µl was analyzed. Upon concluding the experiment, full-length 16S rRNA gene sequencing (as described above) confirmed that the cultures remained axenic throughout the experiment. The maximum growth rate, lag phase length, and carrying capacity were determined through bootstrapped nonparametric spline fits (*n* = 1,000) to the data with *grofit* (v1.1.1-1) while the specific growth rate and generation time were estimated using logistic regression with *Growthcurver* (v0.2.1) (93, 94).

### Phenotypic description of *Ramlibacter aquaticus* sp. nov.

The type strain is LMG 30558^T^. *Ramlibacter aquaticus* strain LMG 30558^T^ did not grow in many of the standard tests used for the biochemical characterization of bacteria, including the API 20 NE microtest system (bioMérieux). However, the biochemical comparison yielded multiple characteristics that allowed us to distinguish it from Ramlibacter solisilvae LMG 30470^T^. *Ramlibacter aquaticus* strain LMG 30558^T^ grew on R2A agar at 35°C and 37°C, but not at 4, 10, 15, or 20°C, while Ramlibacter solisilvae LMG 30470^T^ showed opposite reactions in each of these tests. In addition, phosphoamidase activity (as detected through the API ZYM microtest system [bioMérieux]) was present in the former but absent in the latter. Tyrosine hydrolysis and catalase and oxidase activity were absent in the former and present in the latter.

**(i) Etymology.** L. masc. adj. *aquaticus*, living, growing, or found in or by the water, aquatic.

**(ii) Locality.** Isolated from secondary cooling water of the BR2 nuclear reactor in Mol, Antwerp, Belgium (51.22° N 5.09° E).

**(iii) Properties.** Cells are Gram negative and pleomorphic, with both a coccoid (“cyst-like”) and rod morphology. The overall cell lengths varied between 0.3 and 8 µm; coccoid cells were shorter than 1 µm (0.7 ± 0.2 µm; *n* = 96), and all rod-shaped cells were longer than 1 µm (2.5 ± 1.6 µm; *n* = 70). Grows aerobically in liquid R2A medium at concentrations between 1 to 100 mg liter^−1^ R2A with a lag phase of 20 to 50 h and carrying capacity of 4.3 × 10^6^ cells ml^−1^ to 11.8 × 10^6^ cells ml^−1^, respectively. Exhibits unpredictable and difficult growth on solid R2A medium. White translucent colonies are visible after 7 to 10 days of inoculation at 28°C. Aerobic growth on R2A agar is observed from 25 to 37°C, but not from 4 to 20°C or at 40°C. Optimal growth on R2A agar at 28°C. No growth on nutrient agar, tryptic soy agar, or Luria-Bertani agar medium.

Negative for catalase and oxidase activity. No growth on agar media for testing hydrolysis of Tween 20 or Tween 80. No hydrolysis of esculin or tyrosine. Growth on agar media for testing hydrolysis of starch and casein, but no hydrolysis of either component. No growth in the API 20NE microtest system. Activity of alkaline and acid phosphatase, butyrate esterase (C4), and phosphoamidase is present as detected through the API ZYM microtest system, but no caprylate esterase lipase (C8), myristate lipase (C14), leucyl arylamidase, valine arylamidase, cystin arylamidase, trypsin, chymotrypsin, alpha-galactosidase, beta-galactosidase, beta-glucuronidase, alpha-glucosidase, beta-glucosidase, N-acetyl-beta-glucosaminidase, alpha-mannosidase, and alpha-fucosidase activity. The GC content of the type strain is 70.6 mol%.

### Data availability.

Raw sequence reads are available on NCBI SRA under ID SRP142224. The coassembly and its annotation are available on IMG (https://img.jgi.doe.gov/) under taxon OID 3300014983, MAGs are available from IMG as specified by their taxon OIDs in Table 1, raw sequences of the 16S V3-V4 rRNA gene amplicon surveys are available on NCBI SRA under ID SRP066190. MAGs are also available from DDBJ/ENA/GenBank under accession IDs SAIV00000000 (*R. aquaticus*), SAIW00000000 (*Bacteroidetes* sp. MAG1), and SAIX00000000 (*Bacteroidetes* sp. MAG2). The version described in this paper is version XXXX01000000. Flow cytometry data are publicly available on FlowRepository under ID FR-FCM-ZYFM. The data analysis for this article is available at https://github.com/rprops/Ramlibacter--CW. The *Anvi’o* profile database is available at https://doi.org/10.6084/m9.figshare.6170420, and the *Anvi’o* pangenome analysis is available at https://doi.org/10.6084/m9.figshare.6170117. Supplemental information and figures are available at https://doi.org/10.6084/m9.figshare.7577636. The isolated *R. aquaticus* strain from this study was deposited in the public BCCM/LMG Bacterium Collection (http://bccm.belspo.be/catalogues/) (Ghent, Belgium) under accession number LMG 30558.
