# Correction for Props et al., “Gene Expansion and Positive Selection as Bacterial Adaptations to Oligotrophic Conditions”

**DOI:** 10.1128/mSphere.01143-20

**Published:** 2020-11-25

**Authors:** Ruben Props, Pieter Monsieurs, Peter Vandamme, Natalie Leys, Vincent J. Denef, Nico Boon

**Affiliations:** a Center for Microbial Ecology and Technology (CMET), Ghent University, Ghent, Belgium; b Belgian Nuclear Research Centre (SCK·CEN), Mol, Belgium; c Department of Ecology and Evolutionary Biology, University of Michigan, Ann Arbor, Michigan, USA; d Laboratory of Microbiology, Faculty of Sciences, Ghent University, Ghent, Belgium; e Unit Health, Flemish Institute for Technological Research (VITO), Mol, Belgium

## AUTHOR CORRECTION

Volume 4, no. 1, e00011-19, 2019, https://doi.org/10.1128/mSphereDirect.00011-19. The data availability paragraph in Materials and Methods should read as follows.

### Data availability.

Raw sequence reads are available on NCBI SRA under identifier (ID) or accession no. SRP142224. The coassembly and its annotation are available on IMG (https://img.jgi.doe.gov/) under taxon OID 3300014983, MAGs are available from IMG as specified by their taxon OIDs in Table 1, and raw sequences of the 16S V3-V4 rRNA gene amplicon surveys are available on NCBI SRA under ID SRP066190. The metagenome reconstructed 16S rRNA gene sequence of *R. aquaticus* LMG 30558 is available from NCBI GenBank under reference ID MW131457. The 16S rRNA gene sequence of the *R. aquaticus* LMG 30558T isolate is available from NCBI GenBank under reference ID MW138094. MAGs are also available from DDBJ/ENA/GenBank under accession IDs SAIV00000000 (*R. aquaticus*), SAIW00000000 (*Bacteroidetes* sp. MAG1), and SAIX00000000 (*Bacteroidetes* sp. MAG2). The versions described in this paper are versions SAIV01000000, SAIW01000000, and SAIX01000000. The draft genome assembly of the *R. aquaticus* LMG 30558T isolate is available from NCBI GenBank under ID JADDOJ000000000. Flow cytometry data are publicly available on FlowRepository under ID FR-FCM-ZYFM. The data analysis for this article is available at https://github.com/rprops/Ramlibacter--CW. The *Anvi’o* profile database is available at https://doi.org/10.6084/m9.figshare.6170420, and the *Anvi’o* pangenome analysis is available at https://doi.org/10.6084/m9.figshare.6170117. Supplemental information and figures are available at https://doi.org/10.6084/m9.figshare.7577636. *R. aquaticus* LMG 30558T cultures are available from the BCCM (ID LMG 30558) and DSMZ (ID 110366) culture collections.

